# Tissue Levels of CD80, CD163 and CD206 and Their Ratios in Periodontal and Peri-Implant Health and Disease

**DOI:** 10.3390/cimb44100321

**Published:** 2022-10-10

**Authors:** Mustafa Yilmaz, Esra Demir, Yigit Firatli, Erhan Firatli, Ulvi Kahraman Gürsoy

**Affiliations:** 1 Department of Periodontology, Institute of Dentistry, University of Turku, 20520 Turku, Finland; 2 Department of Periodontology, Faculty of Dentistry, Biruni University, 34010 Istanbul, Turkey; 3 Department of Periodontology, Faculty of Dentistry, Bezmialem Vakif University, 34093 Istanbul, Turkey; 4 Department of Periodontology, Faculty of Dentistry, Istanbul University, 34134 Istanbul, Turkey

**Keywords:** periodontitis, peri-implantitis, macrophage, mannose receptor, scavenger receptor

## Abstract

This study aimed to compare tissue levels of CD80 (pro-inflammatory macrophage-related surface marker), CD163, and CD206 (anti-inflammatory macrophage-related surface markers), and their ratios in periodontal and peri-implant health and disease. Altogether, 36 tissue samples were obtained from 36 participants with clinically healthy gingiva (*n* = 10), healthy peri-implant mucosa (*n* = 8), periodontitis lesions (*n* = 9), and peri-implantitis lesions (*n* = 9). CD80, CD163, and CD206 levels were assessed with immunoblotting. CD163 levels were found to be decreased (*p* = 0.004), and the CD80/CD163 ratio was found to be elevated (*p* = 0.002) in periodontitis lesions compared to healthy gingiva. Peri-implantitis lesions showed a tendency towards a higher CD80/CD163 ratio than in healthy peri-implant mucosa with a borderline difference (*p* = 0.054). No statistically significant difference was detected in CD80, CD163, and CD206 levels of periodontitis lesions when compared to peri-implantitis, and in healthy gingiva when compared to healthy peri-implant mucosa. A disruption in CD80/CD163 balance seems to be related to the pathogenesis of periodontitis and peri-implantitis, being less prominent in the latter. The reason behind this phenomenon may be either suppressed CD163 expression or reduced CD163+ anti-inflammatory macrophage abundance.

## 1. Introduction

Periodontitis and peri-implantitis are chronic inflammatory diseases characterized by progressive bone and attachment loss. Despite having similarities in their clinical appearance, periodontitis and peri-implantitis lesions show different histopathological characteristics [1,2]. Compared to periodontitis lesions, peri-implantitis lesions are approximately double the size, and have larger vascular structures neighboring the inflammatory cell infiltrate and a more ulcerated pocket epithelium [2]. Although plasma cells and lymphocytes dominate both lesions, peri-implantitis sites accommodate more leukocytes and macrophages than those of periodontitis [1]. Moreover, it has been shown that soft tissue biopsies of peri-implantitis lesions contain foreign bodies that are associated with the inflammatory infiltrate [3]. Foreign bodies may induce a prolonged inflammatory state in the host, while macrophages are considered key regulators of the immune-inflammatory response.

Macrophages have diverse roles in host response and homeostasis, such as pathogen recognition, phagocytosis, immune modulation, initiation and resolution of inflammation, and tissue-repair [4]. They can switch their phenotype between the pro-inflammatory M1 and the anti-inflammatory/pro-repair M2 depending on the signals from their environment [5]. Pro-inflammatory macrophage polarization has been associated with various conditions such as rheumatoid arthritis [6], cancer [7], atherosclerosis [8], and also periodontitis [9,10,11,12] and peri-implantitis [13,14]. Macrophage-related surface markers such as CD80, CD163, and CD206 are used in identifying macrophage phenotypes, although macrophages can carry pro- (M1) and anti-inflammatory (M2) surface markers simultaneously [15,16,17]. CD80/CD163 or CD80/CD206 positive cell ratios have been used to immunohistochemically assess macrophage polarization in various conditions such as colorectal cancer [18], arthritis [19], aging [20], lung injury [21] and peri-implantitis [13]. Independent of their role as surface markers of macrophage phenotypes, these proteins contribute to the initiation, progression, and resolution of inflammation with their distinct capabilities: CD80 (pro-inflammatory marker), which is expressed by various immune cells and particularly by antigen-presenting cells, is a co-stimulation molecule activating T-cell functions [22]. CD163 and CD206, on the other hand, have strong anti-inflammatory effects. CD163 is a scavenger receptor for the haptoglobin–hemoglobin complex, which is exclusive to the macrophage/monocyte lineage [23]. CD163 dampens inflammation by clearing hemoglobin and inducing anti-inflammatory cytokine release [24]. CD206, the macrophage mannose receptor, is responsible for endogenous glycoprotein scavenging, pathogen recognition, and antigen presentation [25].

Immunostaining targeting the macrophage-related surface markers of CD80, CD163, and CD206 has been widely used for specifying cell types and subtypes in periodontal and peri-implant tissues [13,14,26,27,28]. Yet, considering the distinct roles of CD80, CD163, and CD206 in immune-inflammatory regulation, it is important to define their total tissue levels in periodontal and peri-implant health and disease. In the present study, we hypothesized that tissue protein levels of CD80, CD163, and CD206, and CD80/CD163 and CD80/CD206 ratios, are dependent on the health status of periodontal and peri-implant tissues. In addition, based on the fact that implants are considered to survive in a chronic low-grade inflammatory state [29], we hypothesized that healthy implants have elevated CD80 levels when compared to healthy gingiva, albeit most likely balanced CD80/CD163 and CD80/CD206 ratios. An increased CD80/CD163 or CD80/CD206 ratio was also expected from peri-implantitis lesions when compared to periodontitis, due to prior research demonstrating that M1 polarization occurs more prominently in peri-implantitis lesions [14]. Therefore, the aim of this study was to compare CD80, CD163, and CD206 levels and their ratios in soft tissue samples of periodontitis, peri-implantitis, clinically healthy gingiva, and clinically healthy peri-implant mucosa, irrespective of the number of cells expressing them.

## 2. Materials and Methods

The conformance of the study to the ethical guidelines of the Helsinki Declaration was approved by the Clinical Research Ethics Committee of Biruni University Medical Faculty, Istanbul, Turkey (2015-KAEK-43-19-27). Since there was no prior research evaluating tissue protein levels of CD80, CD163, and CD206 in periodontal and peri-implant tissues, the sample size was determined according to the participant numbers of two recent studies, in which CD80- and CD206-positive cell counts were investigated by immunohistochemistry [13,14].

The study population was formed of individuals who applied to the Periodontology Department and/or Oral Surgery Department of Biruni University, Istanbul, Turkey between March and December 2021. A single calibrated examiner (E.D.) measured probable pocket depth (PPD), indirect clinical attachment loss (CAL), bleeding on probing (BoP) from six sites of all teeth, and PPD and BoP from all implants with the help of a UNC15 probe (54B XSI, LM Dental, Parainen, Finland). The calibration was conducted with repeated pocket depth measurements of ten patients (intraclass correlation coefficient = 0.87–0.90).

Following clinical examinations, patients diagnosed with generalized Stage III periodontitis [30] or peri-implantitis [31], and periodontally healthy individuals in need of crown lengthening, gingivectomy, tooth extraction, or dental implant application were invited to the study. Inclusion criteria were: (1) willing to participate to the study, (2) being ≥18 years-old, (3) having at least one BoP+ periodontal/peri-implant pocket with a PPD of 6–10 mm for the periodontitis and peri-implantitis groups, (4) having a submerged implant, which is partially exposed and has no visible signs of inflammation for the peri-implant health group, and (5) having no pockets with PPD ≥ 4 mm and having a full mouth score of BoP < 10% for the periodontally healthy group. Exclusion criteria were: (1) having systemic disease, (2) smoking, (3) regular medicine intake, (4) antibiotic or anti-inflammatory medicine use during the 3 months prior to the study, and (5) pregnancy or lactation. According to the criteria above, four study groups (periodontal health (*n* = 10), peri-implant health (*n* = 8), periodontitis (*n* = 9) and peri-implantitis (*n* = 9)) were formed.

One inflamed periodontal/peri-implant pocket (BoP+; PPD of 6–10 mm) of each participant with periodontitis/peri-implantitis was enrolled for sample collection. These samples were obtained at the beginning of the non-surgical therapy with a Gracey curette (American Eagle XP, Missoula, MT, USA), performing a single stroke from the bottom of the pocket until the margin. Healthy peri-implant mucosa samples were collected during the exposure surgery with an incisional biopsy including the mucosa which was in contact with the implant surface. Periodontally healthy samples were obtained from healthy free gingiva (BoP-, PPD ≤ 3 mm) during their corresponding treatment with a crestal incision reaching the bottom of the crevice. All samples were stored in 100 μL PBS (pH 7.2) at −80 °C until they were sent to the Institute of Dentistry, University of Turku, on dry ice for their analyses.

The tissues were ground with a high-speed tissue homogenizer (The TissueLyser LT, Qiagen, Hilden, Germany) and then ultrasonically with 100% amplitude for 10 s (UP50H; Hielscher Ultrasonics, Teltow, Germany). The total protein levels of the samples were measured with a commercial protein determination kit (Pierce BCA Protein Assay Kit, Thermo Fisher Scientific, USA) as specified by the manufacturer. Immunoblot analysis was conducted according to the total protein count of each sample (15 μg protein/well). The same amount of protein for each sample was mixed with Laemmli sample buffer (#1610747; Bio-Rad, Hercules, CA, USA) and mercaptoethanol (#17H1247; Sigma-Aldrich Chemie Gmbh, Steinheim, Germany). Sodium dodecyl sulfate—polyacrylamide gels (8%) were used to separate the proteins. Proteins were then transferred to membranes (Trans-Blot^®^ Turbo™ Transfer System, Bio-Rad, Hercules, CA, USA) and the membranes were incubated with the primary antibodies (1:500 dilution; B7-1/CD80: #AF140, CD163: #AF1607, MMR/CD206: #AF2534; R&D Systems, Bio-Techne, USA) overnight. Then, the membranes were washed and incubated with HRP-conjugated goat IgG antibody (1:1000 dilution; #HAF017; R&D Systems, Bio-Techne, USA). The membranes were treated with the Pierce ECL Plus Western Blotting Substrate Kit (#32106; Thermo Fisher Scientific, Oakwood, OH, USA). The bands were detected by the ChemiDoc MP Imaging System (Bio-Rad, Hercules, CA, USA), and their intensities were analyzed with ImageJ software (National Institutes of Health, Bethesda, MD, USA). The levels of CD80, CD163, and CD206 were recorded as arbitrary density units (AU).

SPSS 27.0.1.0 (IBM, Armonk, NY, USA) was used for the statistical analysis. The difference between groups was evaluated with the Kruskal–Wallis test. The Mann–Whitney U test was used to analyze the sample pairs showing significant differences according to the Kruskal–Wallis test. Qualitative variables were assessed with chi-square. *p* < 0.05 was considered significant.

## 3. Results

The demographic characteristics of the participants and the clinical variables are presented in Table 1. The gender (*p* = 0.552) and age (*p* = 0.822) distribution of the participants were equal across groups. By design, the PPDs of periodontally healthy sites were lower than that of those with periodontitis (*p* < 0.001), while those of peri-implantitis and periodontitis were similar (*p* = 0.811).

**Figure 1:**
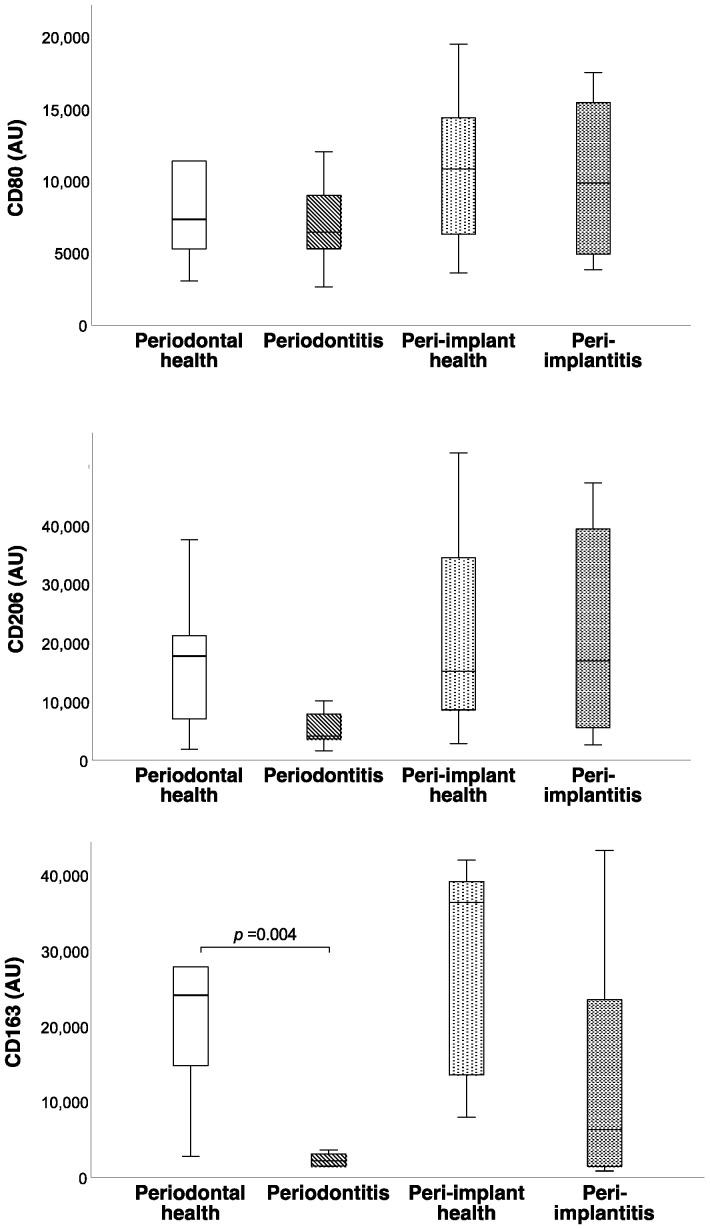
Box-and-whisker diagrams of arbitrary density units (AU) of CD80, CD163, and CD206 levels according to the groups. Multiple comparisons demonstrated statistically significant differences only in CD163 levels, while CD163 levels differed between the periodontitis and periodontally healthy groups but not between the peri-implantitis and peri-implant health groups. Horizontal lines represent the 25th, 50th, and 75th percentiles. *p*-value represents the statistical significance of pairwise comparisons.

**Figure 2:**
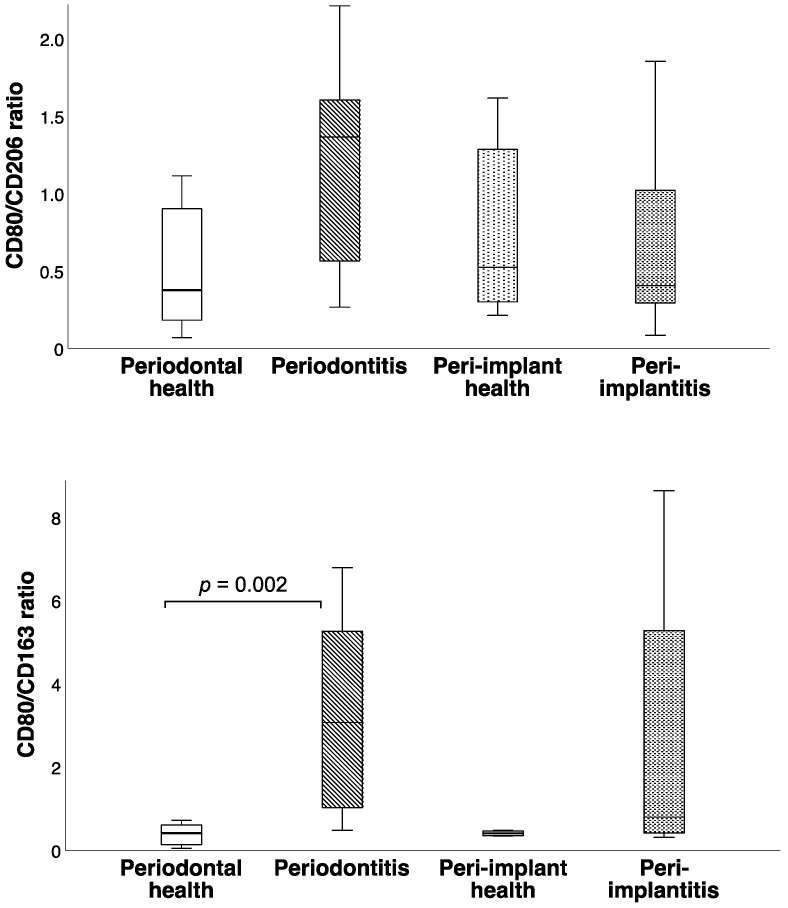
Box-and-whisker diagrams of CD80/CD206, CD80/CD163 ratios according to the groups. The CD80/CD163 ratio was lower in periodontally healthy participants when compared to those with periodontitis, while there was no significant difference between participants with healthy peri-implant mucosa and those with peri-implantitis. The CD80/CD206 ratio was similar among groups. Horizontal lines represent the 25th, 50th, and 75th percentiles. *p*-values represent the statistical significance of pairwise comparisons.

**Figure 3:**
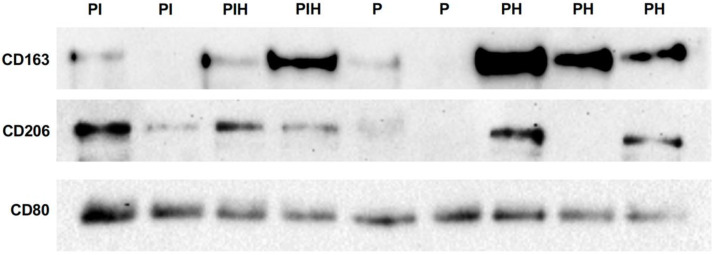
Representative immunoblot images of the analytes according to the groups. Reduced band intensities of CD163 in peri-implantitis and periodontitis lesions can be observed. PI, peri-implantitis; PIH, peri-implant health; P, periodontitis; PH, periodontal health.

Multiple comparison analysis demonstrated statistically significant differences only in CD163 levels (*p* = 0.005) and the CD80/CD163 ratio (*p* = 0.002). Between-group comparisons revealed lower CD163 levels (*p* = 0.004) and a higher CD80/CD163 ratio (*p* = 0.002) in the periodontitis group than in the periodontally healthy group (Figure 1 and Figure 2). A borderline significance (*p* = 0.054) was observed when the CD80/CD163 ratio was compared between peri-implantitis and healthy peri-implant tissue samples, the latter being lower (Figure 2). CD163 levels did not differ between the peri-implantitis and healthy peri-implant groups (*p* = 0.068). No statistical difference was observed in CD163 levels and CD80/CD163 ratio between the periodontitis and peri-implantitis groups (*p* = 0.402, *p* = 0.270, respectively) or between the periodontal health and peri-implant health groups (*p* = 0.594, *p* = 0.859, respectively). Representative tissue levels of CD80, CD163, and CD206 according to immunoblot analyses are presented in Figure 3.

## 4. Discussion

The present study demonstrates an increased CD80/CD163 ratio and decreased CD163 protein levels in tissues affected by periodontitis when compared to healthy gingiva. Periodontitis lesions have been associated with a shift towards an inflammatory macrophage profile [11], but the available information on total tissue levels of macrophage-related surface markers in relation to periodontal and peri-implant status is limited [32]. A decrease in CD163 density was observed in periapical lesions in prior research, while CD163+ macrophage numbers were similar in healthy and inflamed periapical tissues [33]. This indicates that the association between the macrophage cell count (as defined by a specific macrophage surface marker) and the disease can be different to the association between the tissue levels of that specific surface marker and the disease.

In this study, we analyzed the total tissue levels of pro- and anti-inflammatory macrophage-related surface markers and their ratios in relation to periodontitis and peri-implantitis, which was one important novelty. Moreover, the implementation of healthy peri-implant mucosa samples into the study allowed us to profile the macrophage-related surface markers in a non-inflamed peri-implant environment. Naturally, functional implants were not used for sampling sites of the healthy peri-implant mucosa group; instead, mucosa samples of partially-exposed implants were evaluated. These samples were in contact with both the titanium surface and the oral environment, representing healthy functional implants to the utmost. One limitation of our study is its cross-sectional design, which restrains us from revealing a cause–effect relationship. On the other hand, a longitudinal study design including repeated tissue biopsies is not feasible due to ethical considerations. Another limitation of our research is the confined sample size due to the stringent criteria forming the healthy peri-implant mucosa group.

According to our results, the reduced tissue levels of CD163 but not of CD206 are related to periodontitis, which might be on account of their distinct characteristics. Although both CD163 and CD206 are expressed on anti-inflammatory macrophages, they have essential differences in their action mechanisms and in their responses to environmental signals [34,35,36,37]. CD163 binds hemoglobin–haptoglobin complexes and constitutes a defense against hemoglobin-induced oxidative stress [38]. CD163 can also produce anti-inflammatory cytokines, scavenge the tumor necrosis factor-like weak inducer of apoptosis, and recognize bacteria [39,40]. CD206, on the other hand, is associated with endogenous molecule clearance, antigen presentation, cellular modulation, and immune response induction [41]. Our results indicate that the anti-inflammatory, pro-repair nature of CD163 is restrained in tissues affected by periodontitis, which possibly contributes to the disproportionate inflammatory response [24]. This is possibly also associated with the number of anti-inflammatory macrophages during periodontal pathogenesis. CD163 + CD206+ cells are less frequently detected in periodontitis lesions than in healthy tissue samples [28], which is in line with this finding. However, elevated CD163 mRNA expression was also reported previously [42]. It would appear that either CD163 mRNA is not translated to the protein form or the translated protein is degraded rapidly due to the high proteolytic activity in tissues affected by periodontitis.

The decreased levels of CD163 in our study were more prominent in periodontitis lesions than in peri-implantitis lesions. This finding possibly reflects the differences in the proportion of immune cells in the inflammatory cell infiltrate [1] and macrophage polarization [14] between peri-implantitis and periodontitis lesions. The inflammatory cell infiltrate extends more apically and the proportions of macrophages are relatively higher in peri-implantitis lesions [1]. This result may also be due to a slightly elevated CD163-positive macrophage count in peri-implantitis lesions when compared to periodontitis, considering that potentially foreign materials such as excess cement often accompany peri-implantitis [3] and that mature CD163-positive macrophages are involved in foreign body reactions [43]. The tissue levels of CD80 in our study, on the other hand, did not differ between healthy and inflamed gingiva/peri-implant mucosa. CD80 is a co-stimulatory molecule and, together with CD86, they trigger T cell activation, differentiation, and regulation [44]. In gingival tissues, CD80 is expressed on dendritic cells, keratinocytes, B cells, and endothelial cells, but predominantly on macrophages [45]. Because a higher intensity of CD80 expression was demonstrated in diseased sites than healthy sites of the same patients with periodontitis [46], we hypothesized that CD80 levels should increase in periodontitis, which was rejected. This could be due to varying CD80 expression on different cells or CD80-expressing cell counts. To explain, in a prior study, CD80-positive cell percentages were found to not reflect the periodontal condition [45]. Furthermore, different cells expressing CD80 may respond in different ways to various stimuli depending on the circumstances. For instance, no change in CD80 expression level on B cells was reported in periodontitis lesions [47], while a downregulation occurs in CD80 expression on epithelial cells upon challenge with various bacteria and lipopolysaccharides [48]. It has also been shown that pro-inflammatory macrophages respond to *P. gingivalis* challenge with a strong downregulation in CD80 expression, but no dramatic change occurs in anti-inflammatory macrophages [49].

In healthy gingiva, or during the initial stage of gingivitis which is characterized with subclinical inflammation, the cellular infiltrate comprises predominantly neutrophils, but also a low number of lymphocytes and macrophages [50]. Macrophages are the resident immune cells of the periodontium, which can quickly respond to infectious stimuli. In addition to the resident macrophages, more monocytes migrate from circulation to periodontal tissues and differentiate into mature macrophages as a response to increasing inflammation. Macrophages show functional plasticity, which allows them to change their behavior according to their environment [51]. Furthermore, some macrophage subsets which are associated with inflammatory resolution may express both pro- and anti-inflammatory molecules simultaneously [51,52]. Considering that the gingiva is constantly under bacterial challenge, pro-inflammatory macrophage functions such as pathogen recognition, apoptotic cell clearance, and phagocytosis are in balance with anti-inflammatory macrophage functions such as IL-10 and growth factor secretion, promoting cell proliferation, and regeneration. The disruption of this balance towards increased inflammatory burden or incapability to resolve the inflammation may create a chronic inflammatory state. Thus, rather than only the CD80 levels, the CD80/CD163 ratio indicating an imbalance between the inflammatory burden and pro-repair mechanisms can be more definitive in periodontal and peri-implant status. An elevated M1/M2 macrophage ratio indicating this imbalance was also demonstrated in prior research, in various other conditions such as cancer or psoriasis [13,18,28,53,54].

We found the CD80/CD206 ratio to be similar across groups, while a disrupted CD80/CD163 balance was observed in patients with periodontitis, possibly corresponding to the increasing pro- to anti-inflammatory macrophage ratios [10,11,28]. The CD80/CD163 ratio was also decreased in the peri-implantitis group but without a statistically significant difference. However, because this significance demonstrated a border-line value (*p* = 0.054), further research could be beneficial, particularly considering that there are no other studies evaluating the total tissue levels of CD80, CD163, or CD206 in peri-implantitis lesions compared to healthy peri-implant tissues. Finally, a thorough description of CD80, CD163, and CD206 levels, and their ratios in the oral fluids of periodontitis and peri-implantitis patients, may allow them to be considered as early markers of these diseases [55].

## 5. Conclusions

Within the limitations of this study, the CD80/CD163 balance seems to be disrupted in periodontitis and peri-implantitis. The more pronounced elevation of the CD80/CD163 ratio and decrease of CD163 levels in periodontitis lesions indicate different immune characteristics in periodontal and peri-implant lesions. Further studies with CD80 and CD163 knock-out animal models can reveal the cause–effect relationship in their direct contribution to periodontal and peri-implant diseases.

## Figures and Tables

**Table 1:** Age, gender and site-specific clinical variables (probing depth (PPD), clinical attachment level (CAL) and bleeding on probing (BoP) values) according to groups.

	Periodontal Health (*n* = 10)	Periodontitis (*n* = 9)	Peri-Implant Health (*n* = 8)	Peri-Implantitis (*n* = 9)	*p*
Age (mean ± SD)	44.0 ± 11.7	44.4 ± 11.4	43.0 ± 18.8	48.7 ± 10.3	0.822
Male %	50%	33.3%	62.5%	33.3%	0.556
PPD (mean ± SD)	1.5 ± 0.71	7.78 ± 0.83	-	7.78 ± 1.09	<0.001
CAL (mean ± SD)	-	8.1 ± 0.88	-	-	-
BoP %	0	100	-	100	-

SD standard deviation.

## Data Availability

This study is registered on ClinicalTrials.gov (NCT05242354).
